# Advanced deep learning-based image reconstruction in lumbar spine MRI at 0.55 T – Effects on image quality and acquisition time in comparison to conventional deep learning-based reconstruction

**DOI:** 10.1016/j.ejro.2024.100567

**Published:** 2024-04-30

**Authors:** Felix Schlicht, Jan Vosshenrich, Ricardo Donners, Alina Carolin Seifert, Matthias Fenchel, Dominik Nickel, Markus Obmann, Dorothee Harder, Hanns-Christian Breit

**Affiliations:** a Department of Radiology, University Hospital Basel, Petersgraben 4, Basel 4031, Switzerland; b Siemens Healthcare GmbH, Magnetic Resonance, Allee am Röthelheimpark 2, Erlangen 91052, Germany

**Keywords:** Lumbar Vertebrae, Magnetic Resonance Imaging, Deep Learning, Image Processing, Computer-Assisted

## Abstract

**Objectives:**

To evaluate an optimized deep leaning-based image post-processing technique in lumbar spine MRI at 0.55 T in terms of image quality and image acquisition time.

**Materials and methods:**

Lumbar spine imaging was conducted on 18 patients using a 0.55 T MRI scanner, employing conventional (CDLR) and advanced (ADLR) deep learning-based post-processing techniques. Two musculoskeletal radiologists visually evaluated the images using a 5-point Likert scale to assess image quality and resolution. Quantitative assessment in terms of signal intensities (SI) and contrast ratios was performed by region of interest measurements in different body-tissues (vertebral bone, intervertebral disc, spinal cord, cerebrospinal fluid and autochthonous back muscles) to investigate differences between CDLR and ADLR sequences.

**Results:**

The images processed with the advanced technique (ADLR) were rated superior to the conventional technique (CDLR) in terms of signal/contrast, resolution, and assessability of the spinal canal and neural foramen. The interrater agreement was moderate for signal/contrast (ICC = 0.68) and good for resolution (ICC = 0.77), but moderate for spinal canal and neuroforaminal assessability (ICC = 0.55). Quantitative assessment showed a higher contrast ratio for fluid-sensitive sequences in the ADLR images. The use of ADLR reduced image acquisition time by 44.4%, from 14:22 min to 07:59 min.

**Conclusions:**

Advanced deep learning-based image reconstruction algorithms improve the visually perceived image quality in lumbar spine imaging at 0.55 T while simultaneously allowing to substantially decrease image acquisition times.

**Clinical relevance:**

Advanced deep learning-based image post-processing techniques (ADLR) in lumbar spine MRI at 0.55 T significantly improves image quality while reducing image acquisition time.

## Highlights

• Advanced deep learning-based image post-processing improves images and reduces acquisition time by 44.4%.
• Superior image quality ratings and contrast ratios with advanced deep learning-based post-processing.
• Use of the advanced deep learning reconstruction algorithms enhances low-field MRI competitiveness.

## 1 Introduction

Lower back pain is the one of the most common physical ailments in modern society. A vast majority of 80% of people will experience back pain at some point in their life and 25% of adults suffer from back pain at any given time. It is the most common cause for absence from work and patients regularly seek medical help resulting in high costs for healthcare and general economy [1]. Subsequently, lower back pain is one of the most common imaging indications in current radiology practice. The timing and extent of imaging for the evaluation of chronic and acute back pain remain controversial. Radiographs are usually the first choice in patients in whom vertebral fractures are a likely differential diagnosis. However, evaluation of surrounding soft tissues, bone marrow and the spinal canal is the realm of Magnetic Resonance Imaging (MRI), which has been established as the gold standard for most spinal imaging indications, given its unmatched soft tissue contrast [2], [3]. Despite its superior diagnostic accuracy compared to X-ray-based modalities, high examination cost and often limited availability of MR imaging remain to be challenging.

Modern low-field MRI systems are less cost intensive and have fewer and less complex infrastructure demands compared to high-field MRI [4], [5]. This new generation of scanners may help to tackle the mismatch between high patient demand and limited MRI hardware availability, while simultaneously improving the economic considerations for MR imaging of the lumbar spine. Another advantage of new generation low-field MRI scanners is the increased patient comfort during examinations due to the larger bore width and lower noise levels, facilitated by its architecture [6]. Especially the former may be valuable in patients plagued by back pain.

A major limitation of previous low-field MRI systems was the relatively poor diagnostic performance due to low signal-to-noise ratios and image resolution. In contrast, modern 0.55 T MRI systems incorporate advances and technical innovations in coil design, parallel imaging techniques, and especially image post-processing algorithms, all adopted from higher-field-strength MRI scanners [7]. Current studies show that the image quality for musculoskeletal imaging is lower but still diagnostic compared to 1.5 T or 3 T [8]. This is demonstrated, for example, by a study carried out by Breit et al. comparing the image quality at 1.5 T with 0.55 T in volunteers [9]. However, imaging at 0.55 T was performed with a longer measurement time compared to 1.5 T. AI-based methods of image reconstruction promise further optimization potential to improve image quality and reduce scan time at the same time [10], [11].

The aim of this study was therefore to explore the capabilities of an advanced deep learning-based image reconstruction algorithm in terms of image quality and image acquisition time in lumbar spine imaging at 0.55 T compared with established image reconstruction techniques already used in clinical routine.

## 2 Materials and methods

Approval for this prospective single center patient study at the University Hospital of Basel was granted by the local ethics committee (BASEC2021–00166). All patients included gave written inform consent to participate in this prospective study.

### 2.1 Study sample

18 consecutive patients referred for lumbar spine imaging and examined on a 0.55 T MRI scanner system between 01/2023–02/2023 were included into analysis (mean age: 62.4±19.6 years, 8 men). No patient was excluded. Referring departments were neurology (n=1), primary care physicians (n=1), spinal surgery (n=8), and the emergency department (n=8). Clinical questions for the imaging examinations were: nerve root compression (n=7), disc herniation or spinal canal stenosis in nonspecific back pain (n=9), follow-up of an osteoporotic burst fracture (n=1) and follow-up imaging after dorsal decompression surgery (n=1).

### 2.2 MR imaging

All patients were examined on a 0.55 T MRI scanner system (Siemens MAGNETOM Free.Max, Siemens Healthineers, Erlangen Germany) operating the XA50A software version. A lumbar spine imaging protocol from clinical routine, based on recommendations from the European Society of Musculoskeletal Radiology (ESSR) and American College of Radiology (ACR) [12], [13], was used for image acquisition. It comprises of T2-weighted Turbo Spin Echo (TSE) sequences in sagittal and axial plane, a T1-weighted TSE sequence in sagittal plane, and a fluid-sensitive Turbo-Inversion Recovery-Magnitude (TIRM) sequence in coronal plane. During a single examination, two image sets comprising of all four sequences each were first acquired and reconstructed with a conventional deep learning-based reconstruction algorithm (CDLR) and second acquired in accelerated form and reconstructed with an advanced reconstruction algorithm (ADLR). Protocol and pulse sequence parameters are summarized in Table 1.

**Table 1:** Detailed pulse sequence parameters. ADLR = advanced deep learning-based reconstruction, AV = Averages, CDLR = conventional deep learning-based reconstruction, ESP = Echo Spacing, FOV = Field Of View, NEX = Number of Excitations, RA = Reconstruction Algorithm, TE = Echo Time, TI = Inversion Time, TR = Repetition Time, SMS = Simultaneous Multi Slice, ST = Slice Thickness, TF = Turbo Factor.

**Sequence**	**RA**	**TR (ms)**	**TE (ms)**	**TI (ms)**	**ST (ms)**	**Matrix**	**Resolution interpolated (mm)**	**FOV** **(mm)**	**NEX**	**Parallel Imaging**	**TF/ESP (ms)**	**TA** **(ms)**
**T1 TSE sag**	**CDLR**	454	13	-	4	320 ×224	0.5 ×0.5	320 ×320	1	SMS 2	3/13.2	02:28
	**ADLR**	440	13	-	4	320 ×240	0.5 ×0.5	320 ×320	1	GRAPPA 2		01:23
**T2 TSE sag**	**CDLR**	3500	96	-	4	320 ×240	0.5 ×0.5	320 ×320	2	SMS 2, GRAPPA 2	17/13.8	03:23
	**ADLR**	4020	93	-	4	320 ×240	0.5 ×0.5	320 ×320	2	GRAPPA 2		02:06
**T2 TSE ax**	**CDLR**	5910	84	-	4	208 ×156	0.5 ×0.5	200 ×200	3	-	15/14.0	04:51
	**ADLR**	3310	85	-	4	240 ×180	0.5 ×0.5	200 ×300	3	GRAPPA 2		02:39
**TIRM cor**	**CDLR**	4000	69	120	5	304 ×190	0.6 ×0.6	380 ×342	1	SMS 2	15/11.7	03:40
	**ADLR**	6820	93	120	5	304 ×204	0.6 ×0.6	380 ×342	1	GRAPPA 2		01:51

The CDLR algorithm (termed *“Deep Resolve Gain/Sharp”* by the manufacturer) is a product implementation available on the system. It comprises of a denoising algorithm that iteratively enhances conventionally reconstructed images utilizing localized noise information estimated from adjustment data and noise propagation. Furthermore, the resulting images are interpolated using a network based super-resolution algorithm to increase resolution and sharpness. The ADLR algorithm (termed *“Deep Resolve Boost/Sharp”* by the manufacturer) incorporates a deep learning-based raw-data-to-image reconstruction technology. The employed research application uses an architecture inspired by variational networks [14] which generates the image through an iterative process that alternates between data consistency and neural network-based image regularization. For the data consistency pre-calculated coil sensitivity maps are used which are provided to the algorithm beside the raw-data and for the regularization hierarchical down-up networks are chosen[15]. The model parameters were determined by supervised training using about 25,000 fully sampled images acquired with different contrast in different body regions on 1.5 T and 3 T scanners (MAGNETOM, Siemens Healthineers, Erlangen, Germany). The obtained images are then interpolated using the same super-resolution network utilized by the CDLR algorithm. Both algorithms are integrated into the inline image reconstruction of the scanner.

### 2.3 Visual image quality assessment

Visual assessment of all 18 examinations was performed individually by two fellowship-trained, board-certified musculoskeletal radiologists (3 and 2 years of experience). Reading was performed for each patient and each sequence on PACS workstations (Sectra, Sweden). Readers were blinded to patient details, image acquisition parameters and reconstruction technique. Visual assessment was performed in one reading. Subjective evaluations were conducted for all sequences individually to obtain scores for the following parameters: [1] signal/contrast, [2] resolution, [3] assessability of the spinal canal and [4] assessability of the neuroforamina. A 5-point Likert scale was employed for grading: 1 = poor, non-diagnostic image quality, 2 = fair but diagnostic image quality, 3 = moderate image quality, 4 = good image quality, 5 = excellent image quality.

**Fig. 1:**
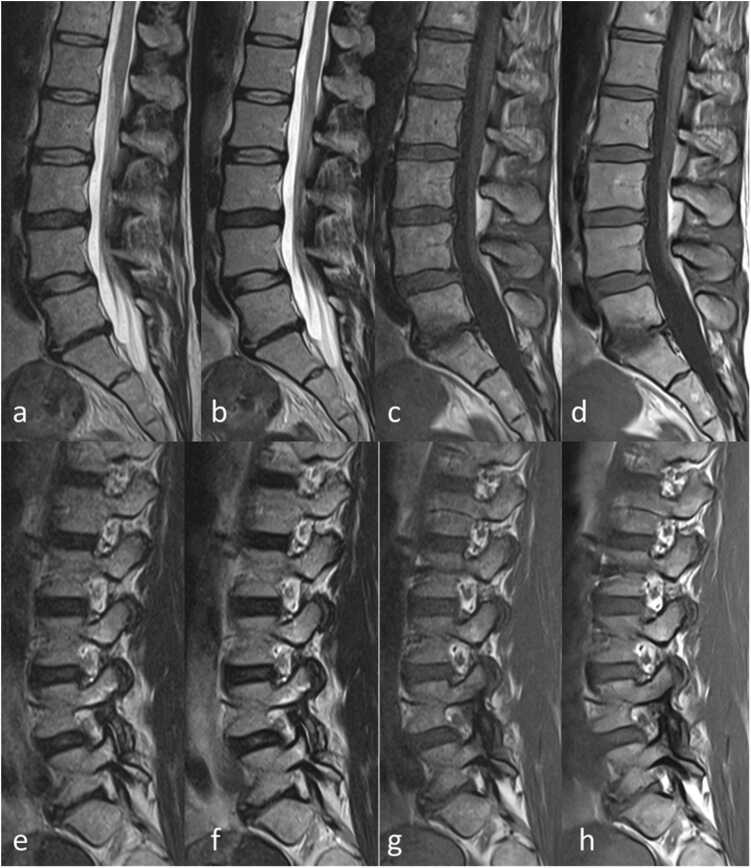
Sagittal T2-weighted (a, b, e, f) and T1-weighted (c, d, g, h) images of the lumbar spine in saggital plane with conventional deep learning-based (a, c, e, g) and advanced deep learning-based (b, d, f, h) image reconstruction.

**Fig. 2:**
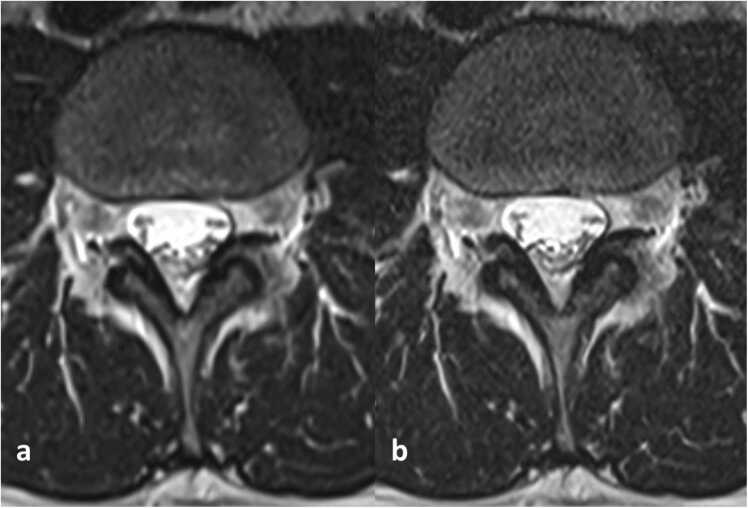
Axial T2-weighted images at the L3 level. with conventional deep learning-based (a) and advanced deep learning-based (b) image reconstruction.

Scoring for signal/contrast was based on the visually perceived contrast between main anatomic structures. This was usually the contrast between spinal cord, cerebrospinal fluid and intervertebral discs for T2 weighted sequences. For T1 weighted sequences, the contrast between bone, perineural fat and the nerve roots. For the evaluation of the resolution, the delineation of the smaller structures (especially the nerve roots in the neuroforamen) was relevant. Assessability of the neural foramina was evaluated on sagittal T1- and T2-weighted sequences, as shown in Fig. 1, as well as on axial T2-weighted images. Tissue contrast between the nerve root and perineural fat in the neural foramen was assessed as illustrated in Fig. 2 [16]. Spinal canal assessability was evaluated on T2-weighted sequences in both axial and sagittal plane. Focus was put on contrast and not the distinct grading of foraminal or spinal canal stenosis.

**Fig. 3:**
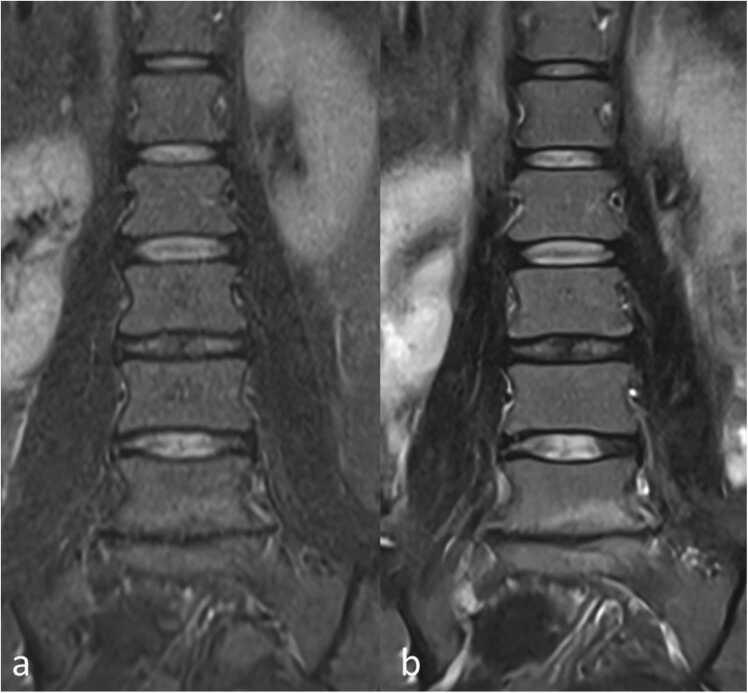
Coronal TIRM images of the lumbar spine in Coronal plane with conventional deep learning-based (a) and advanced deep learning-based (b) image reconstruction.

For coronal TIRM images, both CDLR and ADLR image reconstruction techniques were evaluated regarding signal/contrast and resolution, as illustrated in Fig. 3.

### 2.4 Quantitative image quality assessment

In addition to the visual assessment, a quantitative approach to compare differences in signal intensities between CLDR and ADLR processed sequences was performed by region of interest (ROI) based signal intensity (SI) measurements on all sequences.

ROIs were placed in five different tissues and mean signal intensity values noted: [1] vertebral bone (SI_Bone_), [2] disc (SI_Disc_), [3] spinal cord (SI_Cord_), [4] cerebrospinal fluid (SI_CSF_) and [5] autochthonous back muscle tissue (SI_Muscle_). A relatively homogeneous region of the respective tissue was carefully chosen. All measurements were performed at the level of the conus medullaris at the exact same location for both CDLR and ADLR sequences to ensure consistency. Due to the use of parallel imaging during image acquisition, the following contrast ratios were calculated instead of a basic signal-to-noise ratio calculation [17]:

(1) $${\mathrm{CR}}_{1}=\frac{{\mathrm{SI}}_{\mathrm{CSF}}}{{\mathrm{SI}}_{\mathrm{Cord}}}\mathrm{on sagittal T}2-\mathrm{weighted images and coronal TIRM images}.$$

(2) $${\mathrm{CR}}_{2}=\frac{{\mathrm{SI}}_{\mathrm{Bone}}}{{\mathrm{SI}}_{\mathrm{Disc}}}\mathrm{on sagittal T}1-\mathrm{weighted images}.$$

(3) $${\mathrm{CR}}_{3}=\frac{{\mathrm{SI}}_{\mathrm{Bone}}}{{\mathrm{SI}}_{\mathrm{Muscle}}}\mathrm{on sagittal T}1-\mathrm{weighted images}.$$

### 2.5 Statistical analysis

Statistical analyses were performed in SPSS 26.0 software (IBM Corp., Armonk, NY). Interrater agreement was assessed using the Intraclass Correlation Coefficient (ICC) with a two-way mixed model with absolute agreement. ICC values were interpreted as: poor (<0.50), moderate (0.50–0.75), good (0.75–0.90), and excellent (>0.90) [18].

Mean was calculated from the Likert-Scores, both overall and by sequence. Statistical differences between Likert scores were determined with the Mann-Whitney U test. P-values <.05 were considered to represent a statistically significant difference.

## 3 Results

### 3.1 MR imaging

All sequences were successfully acquired and reconstructed both with the CDLR and ADLR algorithm in all 18 patients. The following diagnoses could be made based on the MRI: subdural hygroma (n=1), high-grade spinal canal stenosis (n=3), known lumbar vertebral burst fracture (n=1), postoperative epidural hematoma (n=1), and Modic II endplate changes (n=2).

Image acceleration and reconstruction using the advanced deep learning-based algorithm reduced imaging time by up to 50% per sequence compared with imaging and reconstruction using the conventional deep learning-based reconstruction algorithm. For example, the acquisition time of the axial T2 weighted sequence was reduced by 45% from 04:51 min (CDLR) to 02:39 min (ADLR). Overall acquisition time of the entire lumbar spine imaging protocol including all four sequences was reduced by 44.4% from 14:22 min to 07:59 min (p<.001) by accelerating image acquisition and using the ADLR technique. Details are provided in Table 1.

### 3.2 Visual image quality assessment

Overall Likert scores from visual assessment by the two readers were higher for images reconstructed with the advanced deep learning-based algorithm (ADLR), compared with images reconstructed by the conventional algorithm (CDLR). This was true for all four parameters, including signal / contrast (4.4±0.5 vs. 3.9±0.4; p<.001), resolution (4.1±0.5 vs. 3.5±0.5; p<.001), assessability of the spinal canal (4.3±0.5 vs. 3.7±0.4; p<.001) and assessability of the neural foramen (4.2±0.5 vs. 3.8±0.3; p<.001). Overall Likert score are summarized in Table 2.

**Table 2:** Comparison of overall Likert scores (range: 1 = non-diagnostic to 5 = perfect quality) from visual image quality assessment between image reconstruction algorithms. ADLR = advanced deep learning-based reconstruction, CDLR = conventional deep learning-based reconstruction. P-values marked with an asterisk (*) represent a statistically significant difference.

**Parameter**	**ADLR**	**CDLR**	***p***
**Signal / Contrast**	4.4±0.5	3.9±0.4	<.001*
**Resolution**	4.1±0.5	3.5±0.5	<.001*
**Assessability Spinal Canal**	4.3±0.5	3.7±0.4	<.001*
**Assessability Neural Foramen**	4.2±0.5	3.8±0.3	<.001*

Detailed analysis of the distinct sequences yielded similar results for most sequences. Highest scores for image signal / contrast were observed for ADLR reconstructed sagittal T1-weighted images (4.7±0.4 vs. 3.9±0.3; p<.001) and coronal TIRM images (4.5±0.4 vs. 3.6±0.4; p<.001). Similarly, resolution was rated highest on these two sequences with ADLR reconstruction (T1 sag: 4.3±0.5 vs. 3.7±0.4; p<.001; TIRM cor: 4.4±0.5 vs. 3.4±0.5; p<.001). Assessability of the spinal canal was rated as almost perfect for ADLR images on sagittal T1-weighted (4.6±0.5 vs. 3.9±0.3; p<.001) and T2-weighted (4.7±0.4 vs. 4.0±0.3; p<.001) sequences. Assessability of the neural foramen was rated best on sagittal T1-weighted ADLR reconstructed images (4.8±0.4 vs. 3.9±0.3; p<.001).

For axial T2-weighted images, ratings were slightly higher for all four parameters when ADLR was used instead of CDLR, though not statistically significant (range: p=.05 −.63). Detailed results per sequence type and reconstruction mode are summarized in Table 3.

**Table 3:** Detailed comparison of Likert scores (range: 1 = non-diagnostic to 5 = perfect quality) per sequence from visual image quality assessment between image reconstruction algorithms. ADLR = advanced deep learning-based reconstruction, CDLR = conventional deep learning-based reconstruction. P-values marked with an asterisk (*) represent a statistically significant difference.

**Parameter**	**Sequence**	**ADLR**	**CDLR**	***p***
**Signal / Contrast**	**T1 sag**	4.7±0.4	3.9±0.3	<.001
	**T2 sag**	4.2±0.2	3.8±0.4	.003*
	**T2 ax**	3.7±0.5	3.6±0.4	.63
	**TIRM cor**	4.5±0.4	3.6±0.4	<.001*
**Resolution**	**T1 sag**	4.3±0.5	3.7±0.4	<.001*
	**T2 sag**	3.9±0.3	3.5±0.5	.01*
	**T2 ax**	3.6±0.4	3.3±0.5	.05
	**TIRM cor**	4.4±0.5	3.4±0.5	<.001*
**Assessability Spinal Canal**	**T1 sag**	4.6±0.5	3.9±0.3	<.001*
	**T2 sag**	4.7±0.4	4.0±0.3	<.001*
	**T2 ax**	4.0±0.4	3.8±0.4	.09
**Assessability Neural Foramen**	**T1 sag**	4.8±0.4	3.9±0.3	<.001*
	**T2 sag**	4.2±0.3	3.7±0.3	.006*
	**T2 ax**	4.0±0.3	3.8±0.3	.12

### 3.3 Interrater agreement

Moderate to good interrater agreement was observed for evaluation of signal/contrast (ICC: 0.68, CI: 0.54–0.79) and resolution (ICC 0.77, CI: 0.69–0.83). Interreader agreement was moderate regarding the assessability of the spinal canal (ICC: 0.55, CI: 0.37–0.69) and poor regarding the assessability of the neural foramen (ICC: 0.37, CI: 0.20–0.52).

### 3.4 Quantitative image quality assessment

For coronal TIRM images, the SI_CSF_ / SI_Cord_ contrast ratio was higher for ADLR-reconstructed images compared with CDLR-reconstructed images (mean: 3.01±0.61 vs. 1.85±0.31; p<.001). For all other contrast ratios, there were no statistically significant differences between ADLR- and CDLR-images. Especially, no statistically significant decreases in contrast ratios were observed, despite acquisition times of ADLR-reconstructed T1- and T2-weighted images being up to 50% faster than those reconstructed with the conventional deep learning-based algorithm. Results for all contrast ratios are summarized in Table 4.

**Table 4:** Comparison of contrast ratios between image reconstruction algorithms. ADLR = advanced deep learning-based reconstruction, CDLR = conventional deep learning-based reconstruction. *P*-values marked with an asterisk (*) represent a statistically significant difference.

**Parameter**	**ADLR**	**CDLR**	***p***
**SI**_**CSF**_**/SI**_**Cord**_**T2 sag**	3.09±0.55	2.98±0.47	.52
**SI**_**CSF**_**/SI**_**Cord**_**TIRM cor**	3.01±0.61	1.85±0.31	<.001*
**SI**_**Bone**_**/SI**_**Disc**_**T1 sag**	1.52±0.37	1.79±0.45	.10
**SI**_**Bone**_**/SI**_**Muscle**_**T1 sag**	1.34±0.30	1.40±0.50	.63

## 4 Discussion

The aim of this prospective study was to investigate how the use of a new advanced deep learning-based image reconstruction algorithm (ADLR) would affect image quality and image acquisition time of 0.55 T low-field MRI of the lumbar spine. Compared with an existing conventional deep learning-based reconstruction (CDLR) algorithm currently used in clinical practice, overall visually perceived image quality of ADLR-reconstructed images was rated superior in all aspects by two fellowship-trained musculoskeletal radiologists (e.g. signal/contrast: 4.4±0.5 vs. 3.9±0.4; p<.001). This was true for all sequences of the imaging protocol, though no statistically significant differences were seen for axial T2-weighted images. Additional quantitative assessment showed a higher contrast ratio for coronal TIRM images (3.01±0.61 vs. 1.85±0.31; p<.001) and unchanged contrast ratios for sagittal T1- and T2-weighted images, despite a 44% reduction in overall image acquisition time (07:59 min vs. 14:22 min) could be achieved by using the ADLR algorithm.

Low-field imaging of the lumbar spine has been subject to research since the first generation low-field MRI scanners were introduced to the market. However, challenges imposed by previous MRI scanner generations prevented the broad use in clinical practice. These challenges included low signal-to-noise ratios and long image acquisition times at field strengths below 1 T[19], [20], [21]. Given the enormous technological progress in MRI hardware (e.g. gradient systems and coils) and software (e.g. parallel imaging and deep learning-based reconstruction techniques) since then, low-field MRI scanners are experiencing a renaissance with many of these innovations being adopted from higher-field MRI systems [22]. Especially with regard to lumbar spine imaging, several recent investigations provided evidence that modern low-field MRI is capable to provide diagnostic image quality compared with 1.5 T MRI [8], [9]. These align well with the results of our study.

Despite image acquisition times in these studies were clinically feasible, without applying deep learning reconstruction algorithms they were still up to four times longer than at 1.5 T (24 min vs. 6 min)[7], [23]. Especially for high-volume practices, this would impose the need to install more than one MRI unit to achieve the same patient throughput. The resulting indirect costs would diminish or even offset the advocated economic benefits of modern low field MRI scanners [5], [7] To render low-field MRI not only competitive by means of image quality, a substantial decrease in acquisition time is needed. Image acceleration in more than one direction, e.g. the combined use of parallel imaging and simultaneous multislice acquisition, is a feasible way to achieve this goal since acceleration factors multiply with each other. To maintain image quality, recently introduced deep learning-based reconstruction algorithms compensate for the undersampling of raw data. For joint imaging, up to 10-fold acceleration in examination time using this technique have been described [24]. In our study, we could achieve a 44% reduction in image acquisition time by using acceleration techniques and an advanced deep learning reconstruction algorithm. The resulting acquisition times of 01:23–02:39 min per sequence and 07:59 min for the entire imaging protocol render 0.55 T low-field MRI of the lumbar spine competitive to current imaging protocols at higher field strengths. Fore reference, Sartorreti et al. reported a total scan time of 11:28 min when using parallel imaging at 1.5 T in 2019 [25] and Longo et al. achieved a protocol time of 5:28 min by the help of simultaneous multislice imaging at 3 T in 2022 [26]. The reduction in acquisition time trough deep learning reconstruction in our is comparable to other recently published investigations, e.g. by Bash et al. who reported a 40% reduction in measurement time for lumbar spine MRI while maintaining image quality in a prospective multicenter study [27].

In general, due to the inherently higher signal-to-noise ratios at 1.5 T and 3 T, much higher acceleration factors can be used than 0.55 T low-field MRI. Even though raw data is acquired in a vastly undersampled manner, advanced deep learning reconstruction algorithms still succeed in reconstructing images of good to excellent quality. A recent study by Almansour et al. outlined the potential of using these reconstruction algorithms in lumbar spine imaging at 1.5 T and 3 T, with reported acquisition times of 0:52 min and 0:40 min for sagittal T1 and T2-weighted images at 1.5 T, and 1:03 min and 0:38 min at 3 T in a research setting, respectively [28]. While these may not be matched by low-field MRI, these scanners offer other advantages, e.g. in terms of cost and patient comfort [5], [6].

When applying image acceleration and reconstruction techniques, it is important to evaluate whether and to what extent the used sequences offer potential for acceleration and if image quality or diagnostic performance would suffer from parameter changes. In our study, visually rated image quality was substantially better for the accelerated and ADLR-reconstructed images compared with the CDLR algorithm. Quantitatively, no decreases in contrast ratio were observed for all sequences. While our initial observations foster the idea that diagnostic performance will not be negatively impacted by applying an advanced deep learning reconstruction algorithm, this needs to be confirmed in clinical studies. Similarly, large studies assessing which imaging protocols / indications would fit imaging at what field strength with what amount of image acceleration are needed [29]. Nevertheless, this study aligns with the current narrative that the current advances in deep learning reconstruction is of vast importance to the future of MRI [30].

The study has limitations. The sample size was small and diagnostic performance was not assessed. Larger prospective studies covering a variety of imaging indications and pathologies are thus needed to confirm the absence of negative effects on diagnostic accuracy. The use of parallel imaging limits quantitative assessment. While methods for the evaluation of objective values (such as signal-to-noise ratio) in parallel imaging are still possible with the help of simulations, to our knowledge no standardized methods exist for the use of additional AI-based reconstruction algorithms. The approach used in our study has been previously reported but is of course not an absolute signal-to-noise-ratio measurement [17].

In conclusion, our study demonstrates that the use of advance deep learning-based reconstruction algorithms in lumbar spine MRI at 0.55 T provides good to excellent image quality while simultaneously cutting image acquisition time almost in half. This may render low-field MRI competitive to imaging at 1.5 T in clinical routine in both aspects, however differences in diagnostic performance need to be evaluated in further studies.

## Ethics Statement

This study has been carried out in accordance with The Code of Ethics of the World Medical Association (Declaration of Helsinki) for experiments involving humans. The manuscript is in line with the Recommendations for the Conduct, Reporting, Editing and Publication of Scholarly Work in Medical Journals and aim for the inclusion of representative human populations (sex, age and ethnicity) as per those recommendations. Informed consent was obtained and the privacy rights of human subjects were always observed.

## Funding Statement

No funding has been received for this work. The opinions expressed in this manuscript are solely those of the authors.

## CRediT authorship contribution statement

**Matthias Fenchel:** Software. **Dominik Nickel:** Software. **Alina Carolin Seifert:** Writing – review & editing. **Ricardo Donners:** Writing – review & editing. **Hanns-Christian Breit:** Writing – original draft. **Dorothee Harder:** Supervision. **Markus Obmann:** Writing – review & editing. **Jan Vosshenrich:** Writing – review & editing. **Felix Schlicht:** Writing – original draft.

## Declaration of Competing Interest

The authors of this manuscript have nothing to disclose. The article comprises original data which has not been previously published in another publication.
